# Effect of balloon pulmonary angioplasty on chronic thromboembolic pulmonary hypertension: an assessment of the learning curve in a Japanese university hospital

**DOI:** 10.1007/s12928-024-01076-4

**Published:** 2024-12-30

**Authors:** Naohiro Komura, Teruyasu Sugano, Fumiaki Ono, Mina Nakayama, Toru Suzuki, Noriyuki Kawaura, Junya Hosoda, Masaaki Konishi, Noriaki Iwahashi, Tomoaki Ishigami, Makoto Mo, Kiyoshi Hibi

**Affiliations:** 1 Department of Cardiology, Yokohama City University Hospital, 3-9 Fukuura, Kanazawa-ku, Yokohama, 236-0004 Japan; 2 Department of Cardiovascular Surgery, Yokohama Minami Kyosai Hospital, 1-21-1 Mutsuurahigashi, Kanazawa-Ku, Yokohama, 236-0037 Japan

**Keywords:** Balloon pulmonary angioplasty, Chronic thromboembolic pulmonary hypertension, Complications, Oxygenation improvement, Quality of life

## Abstract

Balloon pulmonary angioplasty (BPA) is an innovative treatment for chronic thromboembolic pulmonary hypertension (CTEPH). We retrospectively examined the clinical outcomes and complications of BPA at Yokohama City University Hospital (YCUH) between 2012 and 2018. In 2012, we began to conduct BPA sessions in 46 patients with inoperable CTEPH; 34 completed the BPA scheme and the follow-up plan. A longitudinal sub-analysis was performed with cohorts 1 and 2 receiving BPA before and after April 2015. Significant improvements in the mean pulmonary arterial pressure, pulmonary vascular resistance, and other parameters were detected after BPA. The total rate of thoracic complications was 25%. Specifically, the increase in SaO_2_ and home oxygen therapy discontinuation rate, and oral riociguat discontinuation rate was significantly higher in cohort 2 (+ 7.7, 75, and 59%) compared to cohort 1 (+ 3.1, 27, and 10%) (*P* < 0.05). Moreover, the need for non-invasive positive pressure ventilation was significantly lower: 0% (cohort 2) vs. 7% (cohort 1) (*P* < 0.05). The BPA sessions conducted at the YCUH resulted in significant improvements in patients with CTEPH. This study demonstrates a clear learning curve regarding the effectiveness of BPA both in normalizing SaO_2_ and facilitating the cessation of home oxygen therapy, as well as in reducing the incidence of severe complications.

**Graphical abstract:**

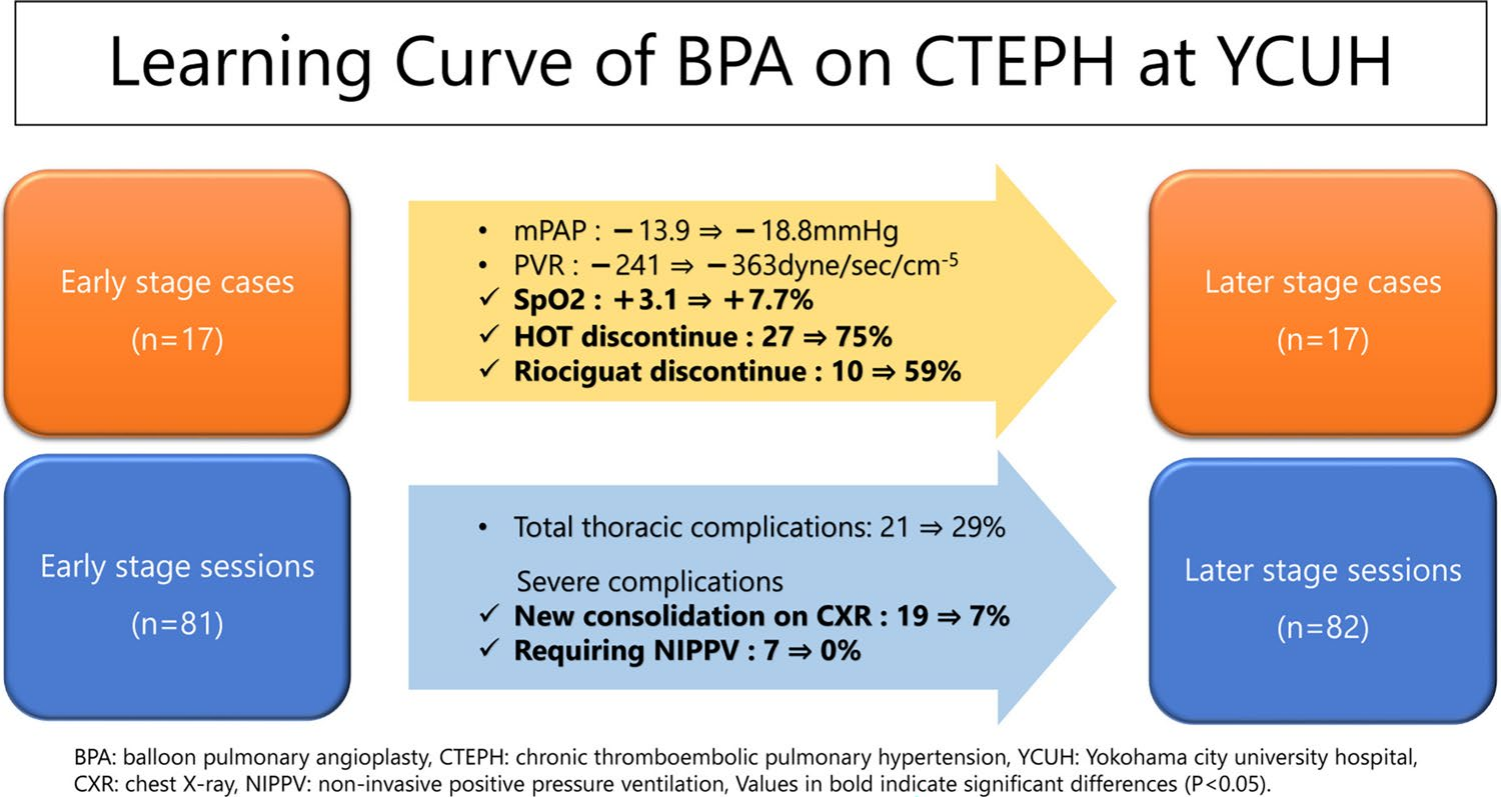

## Introduction

Chronic thromboembolic pulmonary hypertension (CTEPH) is a rare complication of acute pulmonary embolism that leads to progressive pulmonary disease with a fatal outcome if left untreated. In standard clinical settings, CTEPH is defined as any chronic pulmonary thromboembolism case with pulmonary hypertension with a mean pulmonary arterial pressure (mPAP) of at least 25 mmHg. The prevalence of this condition is estimated to range from 0.4 to 9.1% [1].

The pathogeny of CTEPH typically involves non-resolved or incompletely resolved fibro-thrombotic obstructions of the large pulmonary arteries, along with small-vessel vasculopathy in some patients [2]. Although CTEPH diagnosis relies strongly on a history of acute venous thromboembolism (VTE) [3], many patients diagnosed with CTEPH have no history of symptomatic pulmonary thromboembolism, for which it was suggested that other conditions may also result in CTEPH [4]. Both proximal and small-vessel obstruction and pulmonary vascular resistance (PVR) increase in these patients; this results in progressive pulmonary hypertension, right heart failure, and ultimately, death [5, 6].

Pulmonary endarterectomy (PEA) is considered the treatment of choice for operable CTEPH. However, a considerable proportion of patients (approximately 40%) with CTEPH are ineligible for surgery due to the existence of distal lesions or because relevant comorbidities are present [7]. Balloon pulmonary angioplasty (BPA) has emerged as an interesting option for these inoperable patients with CTEPH, alone or in combination with pulmonary hypertension-targeted drugs [8].

BPA is a minimally invasive endovascular procedure intended to widen the obstructed or narrowed pulmonary arteries in patients with CTEPH. To this aim, flexible hollow tubes (catheters) and small balloons are introduced to press blood clots against the pulmonary arteries. In recent decades, this therapeutic approach has been increasingly adopted in many medical facilities worldwide because of its good performance in reducing mPAP in patients with CTEPH, [9] which, in turn, improves hemodynamic parameters and quality of life (QOL).

Nowadays, complication rates tend to decline and hemodynamic goals are almost reachable in Japanese expert centers. However, in hospitals starting with BPA, whether in Japan or other countries, complication rates remain high, and hemodynamic goals are not always achieved.

As observed in most medical interventions, the success of BPA is expected to depend, at least in part, on operator proficiency and expertise. This idea entails the existence of a learning curve for this intervention, a point scarcely addressed to date. According to Fanaroff et al*.* [10], the learning curve and case load are usually relevant factors when assessing complication rates in percutaneous interventional surgery. Also, it has been recognized that those centers reporting their initial experience tend to show poorer results in terms of BPA effectiveness than more experienced ones [7].

Reports on the BPA learning curve are still limited. More real-life data on the outcomes of this novel intervention are required. This retrospective study aimed to report the experience in the use of BPA in patients diagnosed with CTEPH at the Department of Cardiology at Yokohama City University Hospital (YCUH), Japan. The patients were diagnosed between January 2009 and July 2018, and the BPA sessions began in 2012. The effectiveness of BPA was analyzed globally and longitudinally over time to reveal the impact of accumulated experience on the hemodynamic and respiratory outcomes, complication profile, and need for medication and home oxygen therapy (HOT).

## Methods

### Study population and methodology

**Fig. 1:**
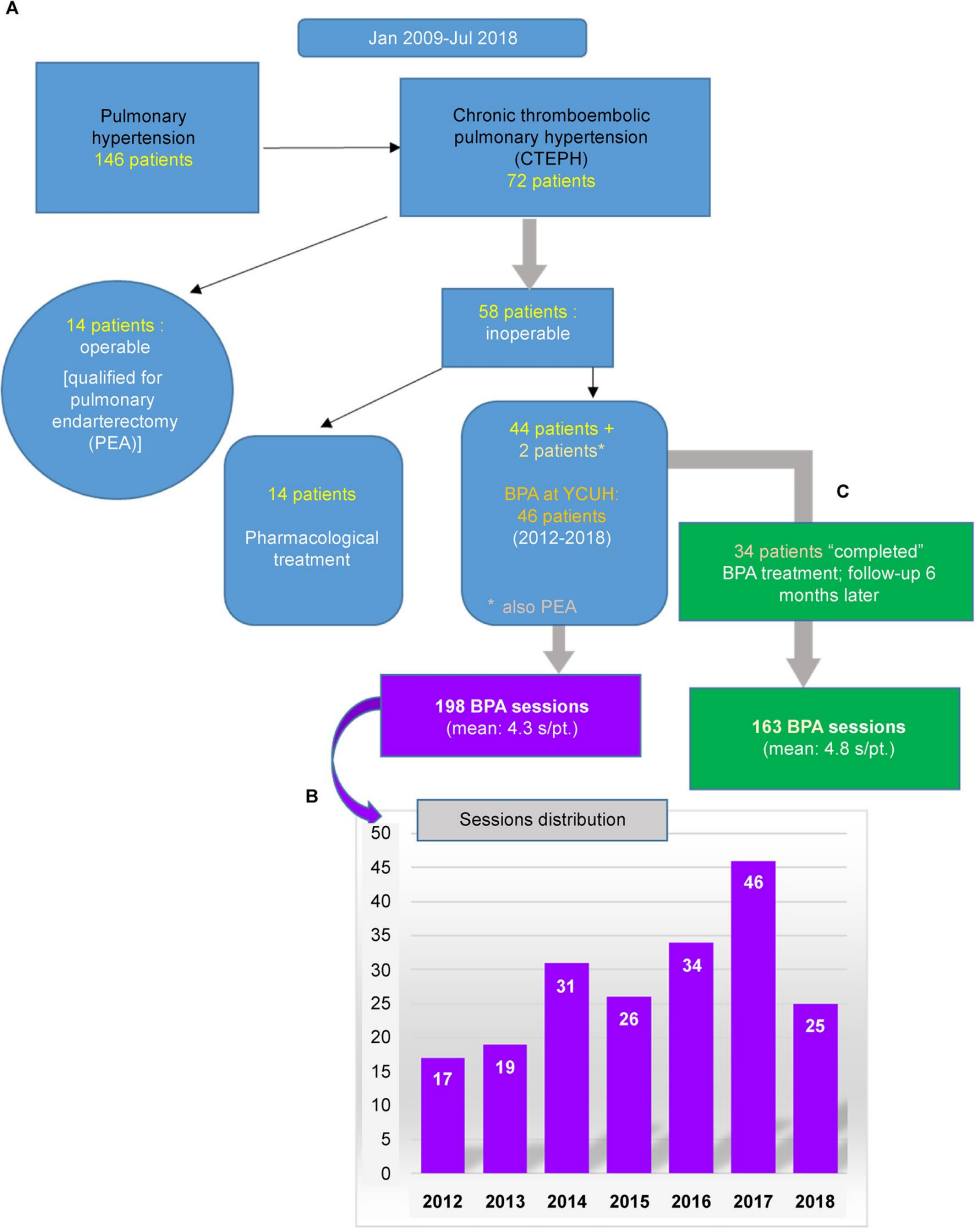
Flowchart of the study and BPA sessions distribution. **a** Between January 2009 and July 2018, 146 patients with pulmonary hypertension were assisted in the Department of Cardiology of the YCUH; 72 were diagnosed with CTEPH, and 58 of them were considered inoperable, for which they were assigned either to medication (*n* = 14) or to BPA (*n* = 44). A total of 46 patients underwent BPA sessions at our institution. **b** 198 BPA sessions were conducted from 2012 to 2018 in these 46 patients; **c** 34 received 163 sessions and completed the follow-up plan. *BPA* balloon pulmonary angioplasty, *YCUH* Yokohama City University Hospital, *s* session; *pt*. patient

Among 146 patients with pulmonary hypertension treated at the Department of Cardiology of YCUH between January 2009 and July 2018, 72 fulfilled the definition of CTEPH. Of these, 58 cases were judged to be inoperable and assigned either to medication exclusively (*n* = 14) or BPA (*n* = 44). Two additional patients received PEA and BPA at our hospital, thus totaling 46 patients (~ 64% of patients with CTEPH) undergoing BPA sessions at the YCUH (Fig. 1a). A total of 198 BPA sessions were conducted from 2012 to 2018 in 46 consecutive patients with CTEPH (mean number of sessions/patient: 4.3) (Fig. 1b), of whom 34 received 163 sessions and completed the BPA sessions and follow-up catheterization plan (mean number of sessions/patient: 4.8) (Fig. 1c). The interventional operator considered the BPA scheme “completed” after 4–6 BPA sessions if there was enough improvement in the hemodynamic parameters and QOL or if all diseased vessels were effectively treated. Right heart catheterization was performed 6 months after the last BPA session to compare the pre- and post-BPA values.

### BPA protocol at our hospital

As the first step in managing patients with pulmonary hypertension, we confirmed the diagnosis of CTEPH through different tests (scintillation, pulmonary angiography, right heart catheterization using a Swan–Ganz catheter). Based on these results, the possibility of PEA was considered and cases of intractable disease were identified. Riociguat was introduced before BPA when the patient’s mPAP was ≥ 30 mmHg; the initial dose was 1.0 mg three times a day (TID) and gradually increased at a rate of 0.5 mg in 2 weeks to reach 2.5 mg TID, according to the drug information. The dose was not increased if side effects, such as hypotension, occurred. Riociguat was discontinued after BPA when the mPAP was < 20 mmHg and the CI was > 2.2 L/min/m^2^. All patients with CTEPH received lifelong warfarin or direct oral anticoagulants (DOACs). HOT was discontinued when SpO_2_ levels were maintained at 95% or higher at rest and 90% or higher during exertion, as measured using a SpO_2_ monitor over several days during hospitalization [11, 12].

The patients were hospitalized for three nights (4 days) to conduct each BPA session. Each patient underwent four to six hospitalizations. In the first and second sessions, BPA was performed using a 2–4-mm-balloon over the entire right/left pulmonary artery area. From the third and fourth sessions onwards, branches of the previous BPA treatments were dilated to optimize the procedure using balloons of appropriate size. The fifth and sixth sessions were mainly conducted to perform BPA in untreated branches, if any.

### Details on the procedure

After the infusion of heparin (100 U/kg), we inserted a 6-French long sheath (Parent Plus, Medikit, Tokyo) into the femoral vein and introduced it into the main pulmonary trunk with a 6-Fr guiding catheter (Mach 1 peripheral IL-3.5, MP, or AL1, Boston Scientific, Natick, MA) using a 0.035-in. wire (Radifocus Guide Wire, Terumo, Tokyo). We crossed a 0.014-in. wire (B-Pahm 0.6, Japan Lifeline, Tokyo, Japan) to the target lesion and dilated the vessel using a balloon catheter of appropriate size with the help of angiographic imaging to determine the size and type of the vessels. Usually, we used a 2.0-mm-balloon for the initial dilatation. Intravascular ultrasound was used for imaging rarely. The brachial or internal jugular veins were sometimes used as access points. The balloon catheters used were Shiden (Kaneka Medical Products, Osaka, Japan) and Crosspander (Japan Lifeline, Tokyo, Japan). Follow-up through right heart catheterization was performed 6 months after the last BPA session.

### Study outcomes

We conducted a comparative analysis of pulmonary hemodynamic and respiratory parameters, including mPAP, PVR, CI, and arterial oxygen saturation (SaO_2_) values obtained by blood gas analysis in a resting supine position during right heart catheterization at room air, 6 MWD, and B-type natriuretic peptide (BNP) levels, before and after BPA. Post-BPA values were determined 6 months after the last BPA session.

Complications typically associated with BPA and HOT/riociguat discontinuation rates were also examined.

### Longitudinal sub-analysis

The 34 patients who completed the treatment and follow-up were classified into two groups to study further how the accumulated experience affected the outcomes and complications of BPA at our hospital: cohort 1, those receiving sessions in the initial stage of our BPA learning curve (first 17 patients and first 81 sessions, before April 2015), and cohort 2, those receiving sessions in a later stage of our BPA learning curve (from patient 18 to patient 34, last 82 sessions, after April 2015). We re-examined the hemodynamic and respiratory parameters and the complications in these early/late patients and session subsets. Both groups were categorized according to the WHO Functional Class (FC) before and after treatment to assess the functional status and response to the intervention.

### Statistical analysis

Data are expressed as mean ± SD (normally distributed variables) or median. Students’ *t* test or one-way ANOVA were used to detect significant differences in continuous variables. Categorical variables are shown as numbers (percentages) and were compared using the chi-square test or Fisher’s exact test. Statistical significance was defined as a two-tailed *P* value < 0.05. All statistical analyses were performed using SPSS version 22 software (IBM Corp., Armonk, NY, USA).

## Results

### Patients with CTEPH: baseline data

Patients with confirmed CTEPH (*n* = 72) were mostly women, with a mean age (± SD) at diagnosis of 63.6 (± 1.1) years (Table 1). Inoperable patients who received BPA treatment were slightly older (particularly those in cohort 2) and had a higher incidence of the most frequent comorbidities: acute pulmonary embolism, dyslipidemia, and hypertension. A small proportion of our patients had coronary artery disease.

**Table 1:** Demographic data and comorbidities of patients with CTEPH diagnosed and treated with BPA at the Department of Cardiology, YCUH, 2009–2018

	Total	BPA and follow-up plan ‘completed’		
	*N* = 72	Global, *n* = 34	Early cohort, *n* = 17	Later cohort, *n* = 17
Age (mean, years)^a^	63.6	65.7	64.4	67.0
Sex				
Male	19 (26)	8 (24)	6 (35)	2 (12)
Female	53 (74)	26 (76)	11 (64)	15 (88)
Acute pulmonary embolism	27 (38)	16 (47)	6 (35)	10 (59)
Dyslipidemia	23 (32)	15 (44)	6 (35)	9 (53)
Hypertension	22 (31)	11 (32)	4 (24)	7 (41)
Mental illness	13 (18)	5 (15)	5 (29)	0 (0)
Malignant tumor	9 (13)	1 (3)	0 (0)	1 (6)
Diabetes	7 (10)	3 (9)	1 (6)	2 (12)
Antiphospholipid antibody syndrome	6 (8)	2 (6)	1 (6)	1 (6)
Coronary artery disease	4 (6)	2 (6)	1 (6)	1 (6)

BPA sessions began in 2012

^a^Except for age, all numerical data are expressed as n, followed by (%)

### Analysis for the entire period

#### Hemodynamic and respiratory indices significantly improved after BPA sessions

**Fig. 2:**
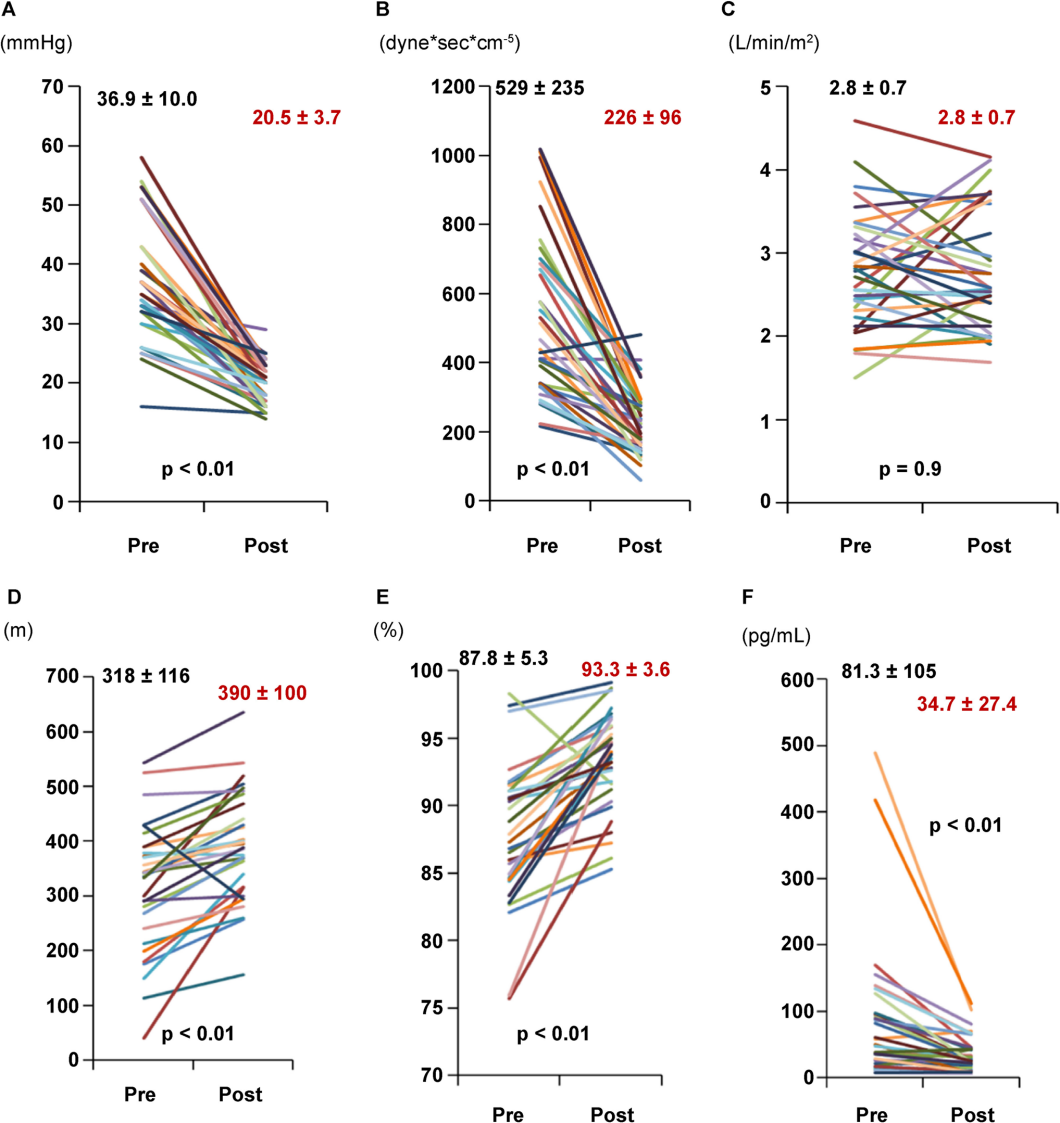
Hemodynamic parameters before and after BPA sessions. Department of Cardiology, YCUH, 2012–2018 (*n* = 34). **a** mPAP: mean pulmonary arterial pressure; **b** PVR: pulmonary vascular resistance; **c** CI: cardiac index; **d** 6 MWD: 6-min walking distance; **e** SaO_2_: oxygen saturation; **f** BNP: B-type natriuretic peptide. Each line depicts changes in individual patients. Mean ± standard deviation values pre-BPA (black) and post-BPA (red) are shown

Figure 2 shows that most patients significantly improved hemodynamic and respiratory parameters after completing the BPA sessions. From a mean pre-treatment value of 36.9 ± 10 mmHg, the mPAP dropped to 20.5 ± 3.7 mmHg, and the PVR to nearly a half: from 529 dyne s/cm^5^ to 226 dyne s/cm^5^; *P* < 0.01 (Fig. 2 a, b). However, the CI remained unchanged (Fig. 2c). Other relevant parameters, such as 6 MWD (Fig. 2d) and SaO_2_ (Fig. 2e), also improved significantly in patients subjected to BPA, who also showed a significant reduction in BNP levels (Fig. 2f). All patients received anticoagulation regimens before the BPA, which were maintained essentially after the intervention: 20 (59%) received warfarin, and 14 (41%) received DOACs.

Among the other outcomes considered in this analysis, we included the assessment of patients receiving HOT and riociguat, a guanylate cyclase stimulator that is useful in the treatment of CTEPH, with insurance coverage for patients with CTEPH in many countries (Table 2). Before starting BPA sessions, most patients were under HOT (91%) and used riociguat (79%). After the BPA sessions, 44% and 47% of the target patients continued HOT and riociguat, respectively. The reduction in these parameters after the BPA sessions was significant.

**Table 2:** Utilization of additional therapies by patients with CTEPH subjected to BPA. Department of Cardiology, YCUH, 2012–2018 (*n* = 34)

	Pre-BPA, *n* (%)	Post-BPA, *n* (%)	*P* value
HOT	**31 (91)**	**15 (44)**	< 0.001
Riociguat	**27 (79)**	**16 (47)**	0.003

Post-BPA values in bold differ significantly from pre-BPA values (*P* < 0.05)

*BPA* balloon pulmonary angioplasty, *HOT* home oxygen therapy

#### Complications

We addressed the frequency and types of complications in our target patients (Table 3). Thoracic complications occurred in 25% of the BPA procedures, with hemoptysis being the most common (22%). These complications were not severe and we were able to manage them easily. Thoracic complications are usually recognized within 48 h after the intervention, and most cases occur within 4–6 h. Bloody sputum and worsening oxygenation are the most common symptoms. New consolidation on chest X-ray (CXR) after BPA was less frequent (13% of BPA sessions). Among these complications, only a few were serious and required non-invasive positive pressure ventilation (NIPPV) or mechanical ventilation, and none of the BPA sessions resulted in patient death.

**Table 3:** Complications associated with BPA sessions (entire period) and as per longitudinal sub-analysis Department of Cardiology, YCUH (*n* = 163)

Complication	No. of BPA sessions associated with complications (%)^a^			
	Entire period	81 early-stage sessions	82 later-stage sessions	*P* value
Total thoracic complications	41 (25%)	17 (21%)	24 (29%)	0.22
Hemoptysis	36 (22%)	14 (17%)	22 (27%)	0.14
New consolidation on CXR after BPA	21 (13%)	**15 (19%)**	**6 (7%)**	**0.033**
Pulmonary artery perforation during BPA	10 (6%)	7 (9%)	3 (4%)	0.19
Severe complications				
Requiring NIPPV	6 (4%)	**6 (7%)**	**0 (0%)**	0.012
Requiring intubation	1 (1%)	1 (1%)	0 (0%)	0.31
Death	0 (0%)	0 (0%)	0 (0%)	

Values in bold indicate significant differences (*P* < 0.05)

*BPA* balloon pulmonary angioplasty, *CXR* chest X-ray, *NIPPV* non-invasive positive pressure ventilation

^a^A total of 163 BPA sessions applied to 34 patients from January 1, 2012, were considered in this analysis

### Longitudinal sub-analysis

#### Hemodynamic and respiratory indices

Table 4 shows the hemodynamic and respiratory parameters before and after BPA sessions in the 34 patients who completed BPA sessions and follow-up, classified into two cohorts: 17 patients who received BPA sessions before April 2015, which may be regarded as assisted patients at an early stage of our learning curve, and 17 patients who received BPA sessions after April 2015, regarded as assisted patients at a later stage of our learning curve, in the context of this longitudinal subanalysis.

**Table 4:** Degree of improvement in hemodynamic parameters and utilization of additional therapies by patients with CTEPH subjected to BPA, longitudinal sub-analysis. Department of Cardiology, YCUH (*n* = 34)

	Before April 2015, 17 early-stage cases			After April 2015, 17 later-stage cases			*P* value
	Pre-BPA	Post-BPA	Change	Pre-BPA	Post-BPA	Change	
mPAP (mmHg)	34.9	21.0	− 13.9	38.8	20.0	− 18.8	0.14
PVR (dyne s/cm^5^)	446	205	− 241	611	248	− 363	0.10
CI (L/min/m^2^)	2.94	3.13	+ 0.19	2.61	2.44	− 0.17	0.14
6 MWD (m)	326	403	+ 77	311	379	+ 68	0.88
SaO_2_ (%)	88.9	92.0	** + 3.1**	86.9	94.6	** + 7.7**	**0.004**
BNP (pg/mL)	59.3	32.9	− 26.4	103.2	36.6	− 66.6	0.17
Rate of change in BNP levels (%)	–	–	20.4	–	–	44.3	0.14
FC I	0	8		0	15		
FC II	12	9		12	2		
FC III	4	0		5	0		
FC IV	1	0		0	0		
Achievement rate of FC I post-treatment (%)	–	47	–	–	88	–	**0.013**
Additional therapies	Pre-BPA	Post-BPA	Discontinue	Pre-BPA	Post-BPA	Discontinue	
HOT	15	11	**4 (27)**	16	4	**12 (75)**	0.006
Riociguat	10	9	**1 (10)**	17	7	**10 (59)**	0.001

Values in bold indicate significant differences (*P* < 0.05)

*BPA* balloon pulmonary angioplasty, *mPAP* mean pulmonary arterial pressure, *PVR* pulmonary vascular resistance, *CI* cardiac index, 6 *MWD* 6-min walking distance, *SaO*_*2*_ oxygen saturation, *BNP* brain natriuretic peptide, *FC* WHO Functional Class, *HOT* home oxygen therapy

While the improvement in SaO_2_ were significantly higher after the BPA sessions applied during the second sub-period compared with the first sub-period (SaO_2_: + 7.7% vs. + 3.1; *P* < 0.05), no significant differences between sub-periods were observed for the rest of the parameters measured including BNP reduction (*P* = 0.17). No statistically significant difference was observed in the rate of change in BNP levels (%) between the two cohorts (*P* = 0.14). Only a trend towards greater effects of BPA on the central hemodynamic indices was detected in the later stage compared with the earlier: change in mPAP: − 18.8 vs. − 13.9 mmHg; change in PVR: − 363 vs. − 241 dyne s/cm^5^.

No significant difference was found in the pre-treatment distribution of WHO FC between cohorts 1 and 2 (*P* = 0.57). However, both cohorts demonstrated significant improvement in WHO FC post-treatment when compared to their pre-treatment statuses. Moreover, when comparing the two cohorts, cohort 2 showed a significantly greater improvement in WHO FC distribution (*P* = 0.01). Notably, the post-treatment achievement rate of WHO FC I was 47% in cohort 1 and 88% in cohort 2 (*P* = 0.013), highlighting a significantly higher success rate in cohort 2.

Considering the need for other therapies or support measures (Table 4), HOT and riociguat use decreased in a notably higher proportion of patients in the second sub-period than in the first sub-period: 75% vs. 27% and 59% vs. 10% discontinued HOT and riociguat, respectively, with significant differences (*P* < 0.05).

#### Complications

According to the Chi-square test, new consolidation on XP and the need for NIPPV decreased at significantly higher rates after BPA sessions in cohort 2 than in cohort 1 (*P* < 0.05) (Table 3). Despite not being significant, it should be noted that pulmonary artery perforation occurred at lower rates during the second sub-period.

## Discussion

BPA has been extensively developed in recent years to offer an option for patients with CTEPH who are ineligible for PEA, the gold standard in managing this condition. This procedure has become a safe and effective strategy for normalizing the mPAP, thus enhancing vital prognosis. However, improving the QOL by withdrawing HOT after achieving sufficient oxygenation should also be a central goal. Several interventional cardiology units worldwide have incorporated BPA in the past few decades and although most reports support its effectiveness, concerns about its risks still exist.

Our results seem to corroborate the concepts expressed in the recent clinical consensus statement of the ESC Working Group on Pulmonary Circulation and Right Ventricular Function, [13] where it was recognized that the hemodynamic positive outcomes reported in a multicenter registry from Japan [14] tended to surpass those reported by the European registries of Poland, [15] France, [16] and Germany, [17] probably because Japanese interventionists have refined the surgical technique for at least 10 years since 2001, developing a more cautious, stepwise approach using 0.014-in. guidewires. However, intrinsic differences between European and Japanese patients with CTEPH have also been reported. There are relevant differences in baseline parameters (e.g., C-reactive protein and fibrinogen concentrations, and red thrombus incidence was higher in European patients). In addition, there were more women than men among patients with CTEPH undergoing BPA in Japan, generally elderly women with less deep vein thrombosis, fewer acute embolic episodes, better cardiac function, and lower arterial oxygen tension [13]. Also, the extended concurrent use of pulmonary vasodilators in Japan may be a relevant factor for better clinical outcomes [13]. In line with the results of the Japanese multicenter registry [14], which included data from seven Japanese institutions performing BPA since 2004, we managed to reduce the mPAP of our patients by ~ 16 mmHg (43% of the baseline value, 47% in the Japanese multicenter registry) and reach an absolute mPAP value of < 25 mmHg. It is worth mentioning that whereas age, sex, and most comorbidities were similarly represented in our cohort and those of the Japanese multicenter registry, our patients had a higher incidence of prior acute pulmonary embolism, mental illness, and malignant tumors. In addition, we succeeded in reducing the PVR and BNP levels and enhancing the 6 MWD and SaO_2_. Corroborating the improved oxygenation levels in our BPA-treated patients, we detected significant discontinuation rates of both HOT and riociguat, which may be linked to the return to normal mPAP levels in many patients.

Regarding complication rates, although lung injury and hemoptysis were somewhat higher than those reported in the Japanese multicenter registry, severe complications requiring intubation were more infrequent (< 1% vs. 5.5%). A meta-analysis from 2021 showed that reperfusion pulmonary edema/injury and pulmonary vascular injury occurred in 25% and 16% of cases, respectively [18].

As a catheter-based procedure, BPA success largely relies on the operators’ technical proficiency, which, in turn, depends on their expertise and experience. The longitudinal sub-analysis presented here suggests that the Japanese BPA technique is so refined that even from the beginning of our learning curve, which began in 2012, we succeeded in decreasing the mPAP to normal levels in most patients, attaining an improved prognosis, a goal that other countries could not achieve yet, as deduced from data reported by 12 European countries (Denmark, Poland, France, Greece, UK, Germany, Spain, Austria, Netherlands, Belgium, Norway, and the Czech Republic) plus the US [13]. Nevertheless, as already indicated, the wider use of pulmonary vasodilators in patients with CTEPH in Japan compared with other countries may have contributed to the higher success of BPA. The procedures performed by more experienced operators at our hospital during the later sub-period (cohort 2) were associated with higher rates of HOT discontinuation and, therefore, improved QOL. A trend towards a lower incidence of severe complications was also observed.

Previous studies indicate that BPA has a steep learning curve. It has been proposed that more than 50 operations must be performed to achieve stable surgical results [19]. In this study, significant improvements in measurable hemodynamic parameters were observed in both cohorts; however, less severe complications were observed in the later cohort. Hong et al. [20] conducted a similar longitudinal analysis, dividing 194 BPA sessions into four groups, and found that the operation time and mean time to dilate one blood vessel decreased steadily, whereas the number of dilated blood vessels per BPA session increased progressively after performing the first 50 procedures. However, consistent with our findings, these authors did not find a significant difference in the degree of change in the mPAP and PVR values when comparing earlier and later procedures.

Nevertheless, because CTEPH is a rare condition, it may be reasonable to centralize BPA procedures in high-volume CTEPH centers to perform a statistically relevant number of BPA sessions, thus allowing intensive operator training, which is expected to reduce the incidence of procedure-related complications**.** In this sense, Jevnikar et al. [21] expressed a similar concept in a recent publication. BPA conducted by more experienced operators is expected to result in improved patient prognosis and better QOL.

There is consensus on the existence of an unavoidable learning curve for BPA, for which most experienced centers usually show significant reductions in the frequency of adverse events in more recently treated patients [22]. For instance, the complication rate fell from 11.2 to 7.7% when comparing the first 1006 sessions with the most recent 562 sessions in the French Reference Centre for Pulmonary Hypertension [16].

In this CTEPH case series, the degree of improvement in mPAP and PVR in the second sub-period was not significantly different from that in the first sub-period. This lack of difference may be because of sufficient reductions in mPAP and PVR obtained since the implementation of BPA at our hospital in 2012. Conversely, other countries have reported modest improvements in mPAP, even at expert centers. The Department of Cardiology at YCUH, which provides a refined BPA procedure following the Japanese style, managed to reduce mPAP to normal levels from the early stage of its learning curve (2012–2015) and allowed normalization of SaO_2_ in the later stage (2015–2018).

The available data indicate sustained hemodynamic benefits and high survival (98.9% at 1, 2, and 3 years) after BPA in Japan [23]. For this reason, BPA has become the strategy of choice for managing patients with inoperable CTEPH in Japan.

In line with the significant progress in SaO_2_, the withdrawal rate of HOT significantly increased in cohort 2 compared with that in cohort 1. Considering that the normalization of pressure precedes the normalization of oxygenation, the effects of BPA may be comprehensively accomplished later in the treatment.

The longitudinal sub-analysis demonstrated that the BPA procedure applied at our hospital was sufficiently refined to result in relevant reductions in mPAP and a low incidence of complications since the beginning of the learning curve. Interestingly, we detected a significant difference in favor of the later period for oxygenation parameters (higher SaO_2_ and HOT discontinuation rates) and less severe complications. In other countries, such as France, there were significant differences between stages of the learning curve regarding mPAP change but little or no significant difference concerning oxygenation or complication rates [7, 16]. For instance, in the study carried out by Piliero et al. in France between May 2013 and February 2020, changes in hemodynamic parameters were greater in the ‘recent period’ (April 2017–February 2020) compared with the ‘initial period’ (May 2013-March 2017): mPAP and PVR decreased by − 37% (vs. − 22%) and − 53% (vs. − 38%), respectively, but the complication rates were not significantly different (5.7% vs. 9.3%) [7].

The rise in pulmonary artery pressure is considered a late event: mPAP is said to increase after 2/3 of the pulmonary artery is obstructed [24]. Therefore, if BPA sessions manage to unblock only 1/3 of the diseased pulmonary artery, mPAP will probably decrease to normal levels, but SaO_2_ will not recover adequately. Therefore, it is important to achieve complete repair of diseased pulmonary arteries through a refined BPA procedure, such as that of the Japanese style, to recover not only normal mPAP but also normal SaO_2_ and better QOL.

One important aspect to note is that in cohort 1, sufficient performance—such as an adequate reduction in mPAP—might have been achieved due to the procedures being performed by CVIT-certified specialists who were already highly skilled in catheter interventions. However, in cohort 2, further improvements were observed, including enhanced FC, improved SaO_2_, withdrawal from HOT, and a reduction in severe complications. These additional improvements suggest that the enhanced outcomes may be attributed to the learning curve effect, reflecting the continuous refinement of technique and increased operator experience over time.

Our findings corroborate that BPA, a minimally invasive intervention, is an effective strategy to treat patients with inoperable CTEPH and improve their QOL. However, there are several points that should be considered while interpreting the findings. It was a single-center study with a retrospective design covering a limited number of patients and BPA sessions.

Another limitation of this study is the procedural transition in the BPA method over time. Beginning in 2016, our institution gradually shifted from the traditional BPA approach—where a small number of branches were treated with a large balloon in the first session—to the more recent Staged Dilatation Strategy. The latter involves dilating multiple branches with a small balloon in the initial session and completing the procedure with a larger balloon in subsequent sessions. However, since the data in this study extend only until 2018, the impact of this procedural transition on the results is likely minimal. Additionally, the findings of this study may not be fully applicable to physicians who are not yet proficient in catheter-based interventions.

In line with nationwide trends, BPA techniques at our institution have evolved over time. As a result, the outcomes of this study may reflect not only the operators’ learning curves but also the overall maturation of BPA treatment in Japan. Considering current treatment strategies as of 2024, it may be possible that even less experienced operators can achieve favorable outcomes if BPA is performed under appropriate guidance. Given that CTEPH is a rare disease, to further improve oxygenation and minimize severe complications, it may be beneficial to refer target patients to specialized centers with experienced BPA teams.

## Conclusions

This study is the first to examine the learning curve for BPA in a Japanese health setting. Through this analysis, we demonstrated that BPA conducted at YCUH since 2012 led to significant improvements in pulmonary hemodynamics, 6 MWD, and BNP levels in patients with inoperable CTEPH.

Normalizing mPAP is an obvious and easily achievable goal in modern Japanese BPA practices. However, only experienced operators can normalize SaO_2_ as an additional but important goal. In patients with CTEPH, the treatment goal is not only to normalize pulmonary arterial pressure and improve vital prognosis but also to normalize SaO_2_ and discontinue HOT to improve QOL. BPA is an important therapeutic option for patients with CTEPH. This study demonstrates a learning curve regarding the effectiveness of BPA in normalizing SaO_2_ and allowing HOT cessation, as well as in minimizing the risk of severe complications.

## Data Availability

The deidentified participant data will not be shared.
